# Research on detection and counting method of wheat ears in the field based on YOLOv11-EDS

**DOI:** 10.3389/fpls.2025.1672425

**Published:** 2025-09-15

**Authors:** Jinai Li, Zongshuai Wang, Xiubin Luo, Bo Feng, Kuijie Gong, Xia Zhang, Jiye Zheng

**Affiliations:** ^1^ Institute of Agricultural Information and Economics, Shandong Academy of Agricultural Sciences, Jinan, Shandong, China; ^2^ Department of Physical Science and Information Engineering, Liaocheng University, Liaocheng, Shandong, China; ^3^ Crop Research Institute, Shandong Academy of Agricultural Sciences, Jinan, China

**Keywords:** wheat ears, object detection, DySample, lightweight, YOLOv11

## Abstract

**Introduction:**

As a major food crop, accurate detection and counting of wheat ears in the field are of great significance for yield estimation. Aiming at the problems of low detection accuracy and large computational load of existing detection and counting methods in complex farmland environments, this study proposes a lightweight wheat ear detection model, YOLOv11-EDS.

**Methods:**

First, the Dysample dynamic upsampling operator is introduced to optimize the upsampling process of feature maps and enhance feature information transmission. Second, the Direction-aware Oriented Efficient Channel Attention mechanism is introduced to make the model focus more on key features and improve the ability to capture wheat ear features. Finally, the Slim-Neck module is introduced to optimize the feature fusion structure and enhance the model’s processing capability for features of different scales.

**Results:**

Experimental results show that the performance of the improved YOLOv11-EDS model is significantly improved on the global wheat ear dataset. The precision is increased by 2.0 percentage points, the recall by 3.5 percentage points, mAP@0.5 by 1.5 percentage points, and mAP@0.5:0.95 by 2.5percentage points compared with the baseline model YOLOv11. Meanwhile, the model parameters are reduced to 2.5 M, and the floating-point operations are reduced to 5.8 G, which are 0. 1 M and 0.5 G lower than the baseline model, respectively, achieving dual optimization of accuracy and efficiency. The model still demonstrates excellent detection performance on a self-built iPhone-view wheat ear datasets, fully verifying its robustness and environmental adaptability.

**Discussion:**

This study provides an efficient solution for the automated analysis of wheat phenotypic parameters in complex farmland environments, which is of great value for promoting the development of smart agriculture.

## 1 Introduction

Wheat is one of the most important food crops globally (Cai et al., 2019), its stable yield and secure supply are directly related to global food security (Wen et al., 2022). Accurate and efficient detection and counting of wheat ears are of great significance for wheat yield estimation and the development of precision agriculture (Zhang et al., 2007). In terms of yield estimation, the number of ears is a key factor determining yield, and its precise counting results can provide a scientific basis for quantitative yield evaluation, thereby supporting grain reserve decisions and market supply-demand regulation (Liu T. et al., 2017; Wang D. et al., 2021). In precision agriculture, detection and counting data can provide decision support for precision fertilization, irrigation, etc., improving resource utilization efficiency and reducing waste and pollution. However, traditional manual counting methods are not only time-consuming and labor-intensive but also their results are easily affected by factors such as subjective experience and fatigue, making it difficult to meet the needs of large-scale agricultural production for data timeliness and consistency (Jin et al., 2017; Xiong et al., 2019). Therefore, developing efficient and accurate automated detection and counting technologies for wheat ears is of great practical significance for promoting the development of smart agriculture.

In recent years, with the rapid development of computer vision and deep learning technologies, object detection techniques have been increasingly applied in the agricultural field (Zhu et al., 2016; Yuan et al., 2022). Among them, object detection algorithms based on Convolutional Neural Networks have become a research hotspot in the field of wheat ear detection due to their ability of automatic feature learning (Alzubaidi et al., 2021). These algorithms can automatically learn features from images to achieve accurate target recognition and localization. In the field of wheat ear detection, many scholars have attempted to use various object detection algorithms, such as RCNN (Bharati and Pramanik, 2020), Faster-RCNN (Liu B. et al., 2017), Mask-RCNN (Lin et al., 2020), and the YOLO series (Vijayakumar and Vairavasundaram, 2024). For example, He et al. (He et al., 2020) improved the YOLOv4 network structure, re-clustered anchors using the k-means algorithm, and proposed a robust wheat ear detection method suitable for natural scenes, which relies on UAV for detection. Khaki et al. (Khaki et al., 2022) developed a WheatNet detection network for wheat ear counting, which significantly reduced model parameters compared to other methods. Wang et al. (Wang Y. et al., 2021) addressed occlusion and overlap issues in wheat ear counting by proposing an improved EfficientDet-D0 model, achieving a counting error rate of only 5.8%. Sun et al. (Sun et al., 2022) constructed a lightweight Wheat Detection Network (WDN) for precise wheat ear detection and counting, with an inference time of 25 ms. Zhou et al. (Zhou et al., 2022) proposed a Multi-Window Swin Transformer network to solve the problem of low detection accuracy under complex field conditions. Bhagat et al. (Bhagat et al., 2021) introduced a lightweight WheatNet-Lite structure, reducing parameters by 54.2M compared to YOLOv3. Shi et al. (Shi et al., 2023) developed an improved lightweight method (YOLOv5s-T) with a model size of only 9. 1M and 2.3 ms reduced inference time. Zang et al. (Zang et al., 2022) integrated an improved attention mechanism into YOLOv5s, achieving 71.61% accuracy in wheat ear counting. Ye et al. (Ye et al., 2023) proposed a lightweight WheatLFANet, achieving an average precision (AP) of 0.9 for complex field wheat ears. Shen et al. (Shen et al., 2023) developed a YOLOv5s-based lightweight method using ShuffleNetV2 for feature extraction, introducing lightweight upsampling to maintain accuracy, with a model weight of 2.9MB. Meng et al. (Meng et al., 2023) proposed YOLOv7-MA, which enhanced feature extraction via convolutional block attention modules and micro-scale detection layers, maintaining high accuracy during both filling and maturity stages.

Despite certain progress in existing studies, limitations remain in complex farmland scenarios. On the one hand, overlapping and occlusion of dense wheat ears, as well as interference from similar backgrounds, easily lead to missed detections or false detections by models, especially insufficient capability to capture small-sized or irregularly shaped ears. On the other hand, some lightweight models sacrifice the depth of feature extraction to control computational load, resulting in reduced robustness in practical environments such as light changes and image blurriness, which makes them unable to meet the real-time detection requirements of field mobile devices. Therefore, how to improve the model’s ability to capture features of wheat ears and resist interference in complex scenarios while ensuring its lightweight nature remains an urgent problem to be solved.

To address the above issues, this paper proposes a wheat ear detection and counting method based on YOLOv11-EDS, which achieves dual improvements in accuracy and efficiency through collaborative optimization of multiple modules. Firstly, the Direction-aware Oriented Efficient Channel Attention mechanism is introduced into the neck network to adaptively enhance the weights of key feature channels of wheat ears, thereby improving the model’s ability to distinguish between targets and backgrounds. Meanwhile, the Dysample upsampling operator is used to replace traditional methods, which preserves more detailed information during the magnification of feature maps and enhances the ability to capture small-sized and dense wheat ears. In addition, the Slim-Neck module is introduced to optimize the feature fusion layer, which streamlines network parameters and reduces computational load while improving the efficiency of feature transmission. The improved model can achieve accurate detection and counting of wheat ears in complex farmland environments, and its lightweight design allows it to be deployed on field mobile devices. This provides efficient technical support for wheat yield estimation and field management, and promotes the intelligent upgrading of agricultural production.

## 2 Materials and methods

### 2.1 Dataset construction

#### 2.1.1 Global wheat head detection dataset 2021

**Figure 1:**
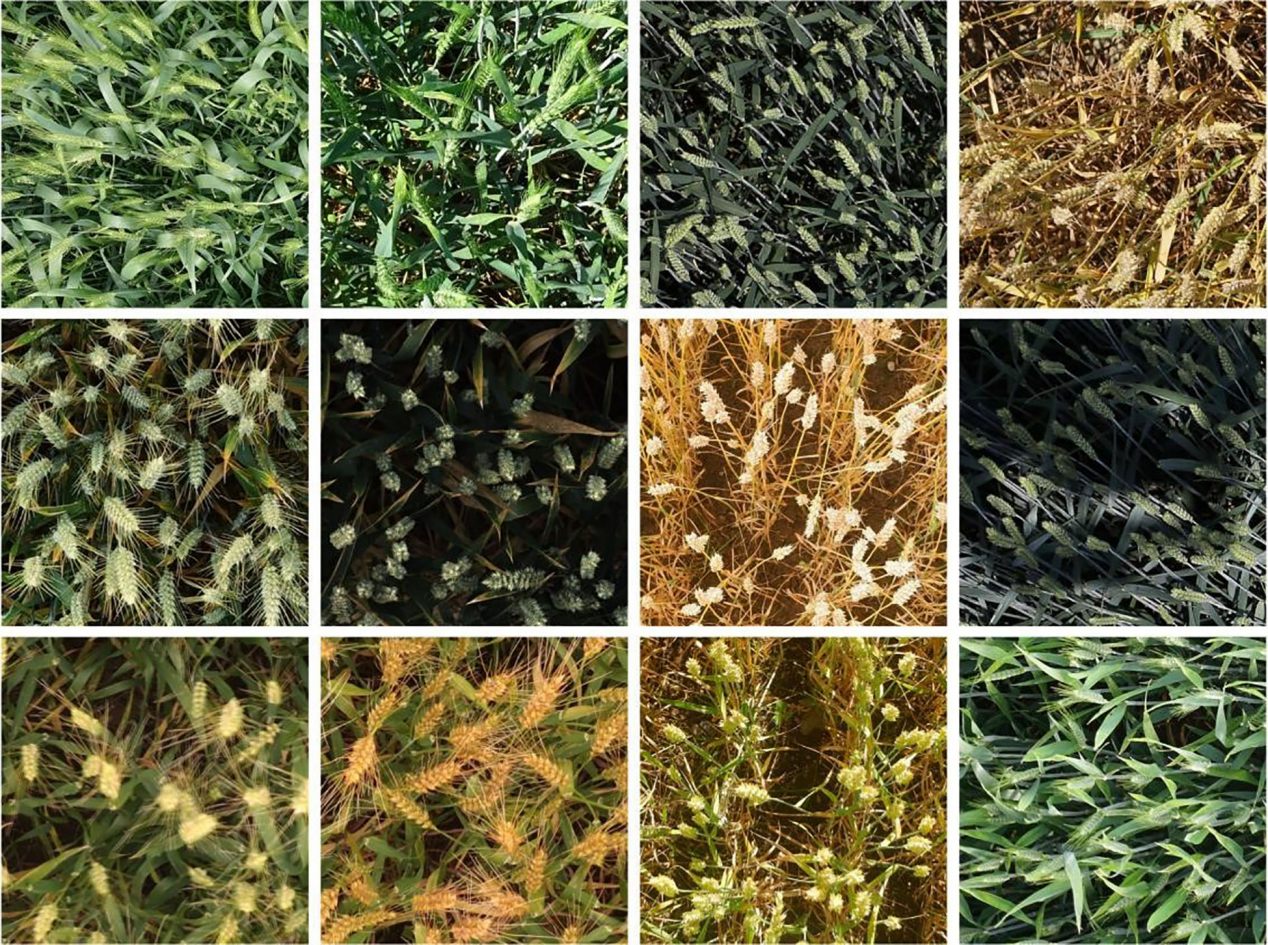
Some example images.

In this study, the Global Wheat Head Detection Dataset 2021 was selected as the benchmark data source (David et al., 2021). Jointly constructed by international agricultural research institutions, this dataset comprises field images captured from major wheat-producing regions across 12 countries/regions, including Europe, Asia, and North America. It contains 6,422 RGB images with a resolution of 1024×1024 pixels, accompanied by 275,187 corresponding bounding box annotations. A subset was constructed by selecting 1,140 images from the GWHD 2021 dataset. The sampling strategy was designed to maximize the visual diversity and complexity of the dataset, ensuring the model’s adaptability to complex field environments. Selection was based on quantifiable features derived from image content itself, with emphasis on achieving broad variation in key parameters such as wheat ear density, scale, occlusion level, and lighting conditions to enhance the model’s generalization capability and robustness. The sampling strategy and detailed label distribution metrics of the subset are presented in **Table 1**, and representative examples of wheat ear images are shown in **Figure 1**.

**Table 1:** Subset sampling strategy and label distribution metrics.

Metric	Details
Total images	1,140
Total annotated ears	40,535
Mean ears per image	35 ± 12 (SD)
Density distribution	10–20 ears: 18%; 21–40 ears: 52%; 41–60 ears: 23%; >60 ears: 7%
Sampling criteria	Based on visual diversity (density, scale, occlusion, lighting)

#### 2.1.2 Self-built iPhone-perspective wheat ear dataset

This dataset was collected at the Comprehensive Experimental Base of Shandong Academy of Agricultural Sciences (117°1’E, 36°7’N) in strict accordance with standardized protocols: image collection was conducted during the wheat filling stage (May 7, 2025) and maturity stage (May 27, 2025) respectively. During the collection process, an iPhone device was used for shooting with fixed parameters: the camera height was uniformly set to 1.2 meters above the ground, and the shooting depression angle was maintained at 15°; the target variety was Jimai 44; each image corresponded to a 1m² standard sampling area to ensure spatial scale consistency. The obtained original images have a resolution of 4284×5712 pixels. Considering the requirements and limitations of model training on computing resources, preprocessing was performed on the collected images. The images were uniformly cropped to 1024×1024 pixels, and images with blur and distortion exceeding the thresholds were excluded according to image quality evaluation standards.After screening, 31 wheat images from the milk stage and 25 from the ripening stage were finally collected.

### 2.2 Data annotation

**Figure 2:**
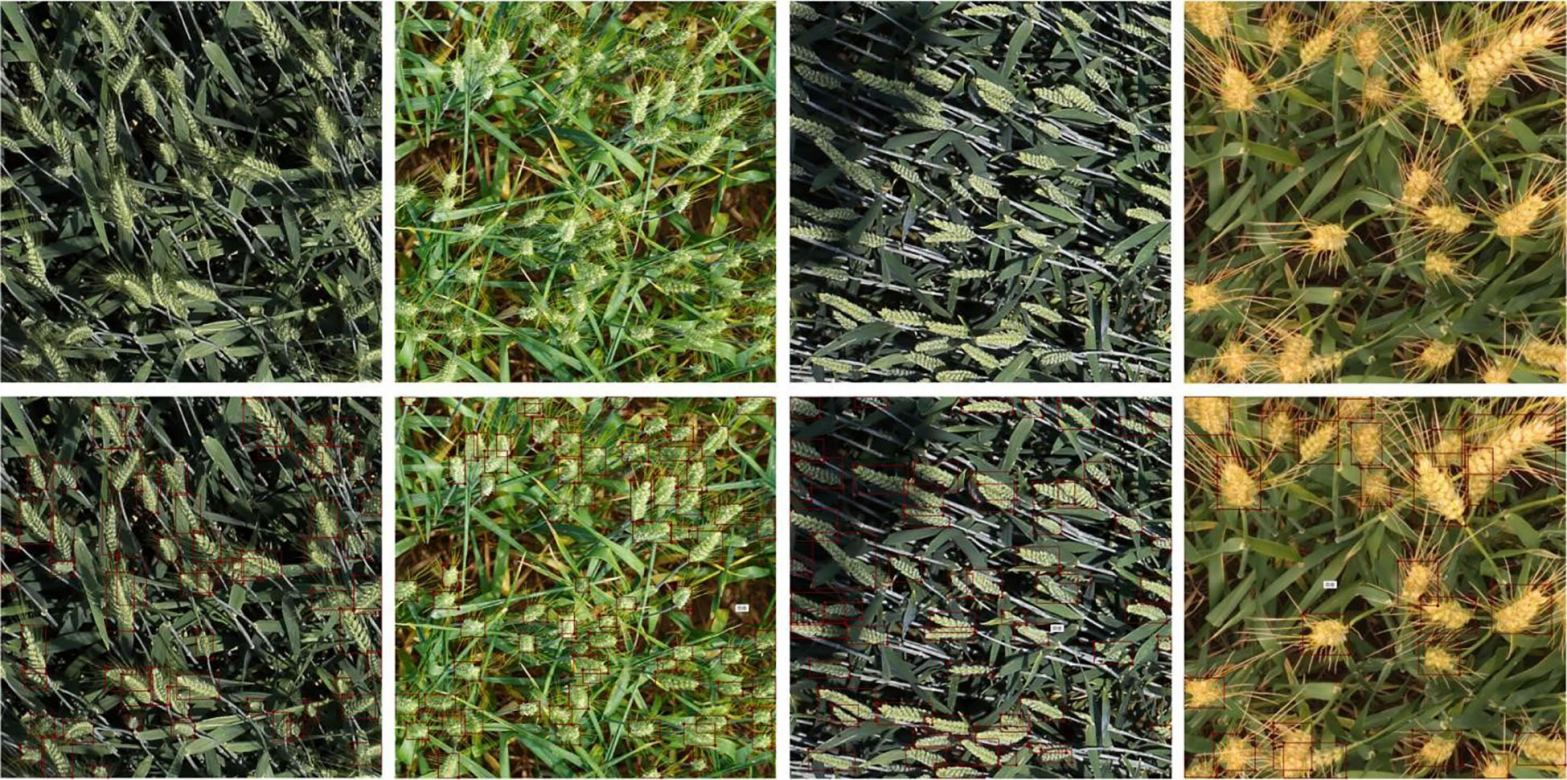
Some examples of annotated images.

The Labelme annotation tool was employed to perform rectangular bounding box annotation on the Global Wheat Head Detection Dataset. Annotated files were saved in JSON format and subsequently converted into TXT format (required for model training), totaling 40,535 labels. During annotation, the category label for wheat ears was defined as “wheat”. Post-annotation, the dataset was partitioned into training, validation, and test sets following an 8:1:1 ratio. Partial annotated images are illustrated in **Figure 2**.

### 2.3 YOLO-EDS

**Figure 3:**
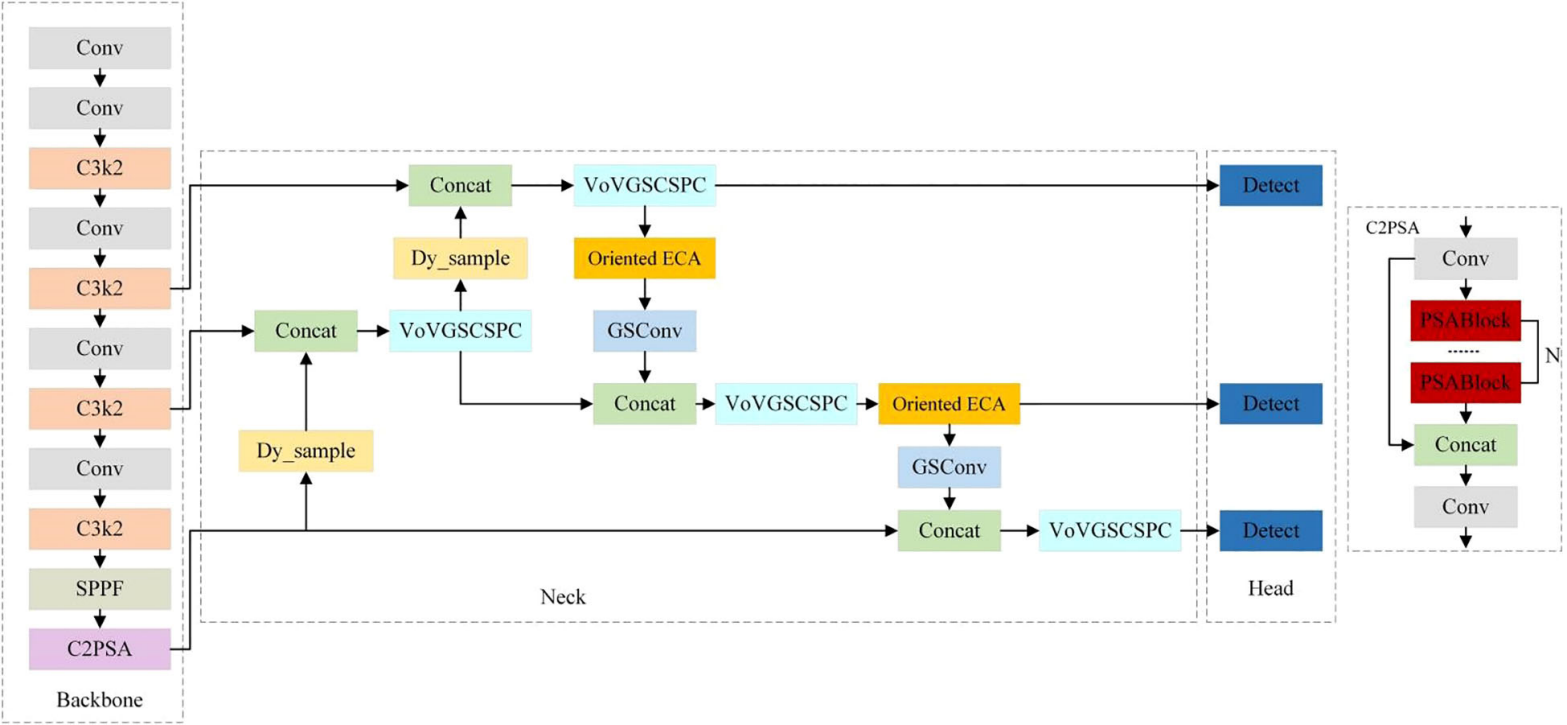
The network structure of YOLOv11-EDS.

YOLOv11, developed by Ultralytics, represents a state-of-the-art real-time object detection model (He et al., 2025). Building upon the core technical framework of the YOLO series, the model achieves comprehensive enhancements in both detection accuracy and efficiency through systematic architectural innovations and performance optimizations. The network architecture of YOLOv11 comprises three key modules: the Backbone, Neck, and Head. These modules closely collaborate and complement each other, enabling object detection via cross-module information interaction (Khanam and Hussain, 2024).While YOLOv11 can detect wheat ears in field scenarios, complex field environments, plant occlusions, and diverse wheat ear morphologies cause conventional feature extraction and fusion methods to struggle in capturing critical features. In particular, the features of small-sized wheat ears are easily overlooked by the network, leading to limited detection accuracy. To address these issues, this study proposes a lightweight network model improved from YOLOv11, termed YOLOv11-EDS, whose structure is illustrated in **Figure 3**.

When an original-resolution field wheat ear image is input, YOLOv11-EDS employs the following improvement strategies for image processing. First, the Dysample upsampling operator is incorporated to better preserve image details while enlarging the feature map, enabling clear presentation of small-scale wheat ear features. Next, the Efficient Channel Attention mechanism is added to allow the model to adaptively focus on key features, effectively suppressing background interference from wheat stalks and leaves during feature extraction, and accurately capturing important feature information of wheat ears across multiple scales. Then, the Slim-Neck module is used to replace the feature fusion layer, realizing long-range feature interaction and local feature interaction for each pixel in the image, and optimizing feature fusion and transmission.

#### 2.3.1 Dysample

In the task of field wheat ear detection, optimizing the upsampling stage is crucial to effectively address the challenges of complex field environments, morphological differences of wheat ears, and scale variations. This study introduces the Dysample dynamic upsampling operator to improve the effect of multi-scale feature fusion and enhance the model’s detection capability for wheat ears of different scales.

**Figure 4:**
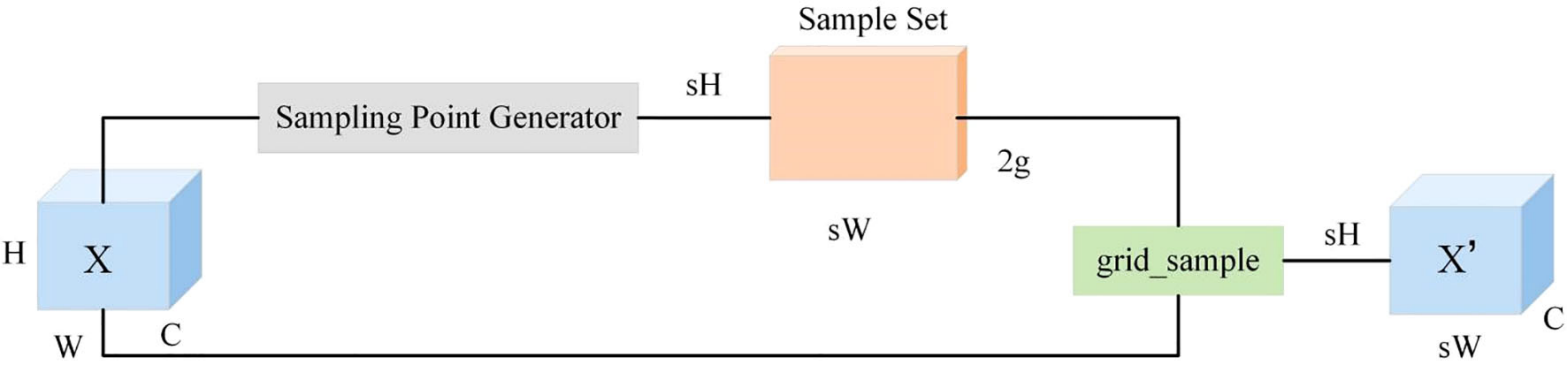
DySample structure diagram.

The Dysample operator (Guo et al., 2024; Lin et al., 2024; Abulizi et al., 2025), drawing on the concept of deformable convolutions, breaks through traditional kernel-based methods. It can dynamically learn the sampling offset for each position based on the semantic distribution of the input feature map, thereby enabling more flexible feature upsampling. Unlike traditional upsampling operators that rely on fixed sampling patterns, the Dysample operator dynamically adjusts the sampling positions according to the content of the input feature map. Taking the PyTorch framework as an example, Dysample mainly adopts point sampling, which not only improves resource utilization efficiency but also simplifies the operator implementation process, providing technical guarantees for the real-time requirements of the detection models. The structure of Dysample is shown in **Figure 4**.

Compared with traditional upsampling methods, the Dysample operator exhibits significant advantages. Traditional upsampling methods tend to lose important detail information when restoring feature map resolution, resulting in feature blurring. In contrast, the Dysample operator can adaptively adjust the sampling positions according to the content of the input feature map, enabling better capture of detailed information in the feature map. In field wheat ear detection scenarios, where wheat ears vary in shape, size, and posture, the Dysample operator can adapt to these variations by learning offsets, allowing sampling points to accurately extract wheat ear features. This enhances the model’s robustness in different environments and improves detection accuracy and stability.

In this study, the Dysample upsampling operator was applied to the Neck network of YOLOv11. The core function of the Neck network is to fuse feature information across different scales, providing richer and more accurate feature representations for subsequent object detection. In the traditional YOLOv11 Neck network, upsampling operations often use methods such as bilinear interpolation, which easily lead to feature information loss. During the feature fusion process in the Neck network, this study replaced the original upsampling method with the Dysample upsampling operator. Specifically, when upsampling low-resolution feature maps to the same size as high-resolution feature maps, the Dysample operator is used for upsampling, thereby better preserving the detail information in low-resolution feature maps and improving the feature fusion effect. During model training, the parameters of the Dysample operator were optimized together with other parameters of YOLOv11. Through the backpropagation algorithm, the model can automatically learn appropriate sampling offsets, enabling the Dysample operator to better adapt to the characteristics of field wheat ear detection tasks.

#### 2.3.2 Direction-aware oriented efficient channel attention

Field wheat ear detection faces numerous challenges. Wheat ears grow in a long strip shape, with dense arrangement, inclined distribution, and mutual occlusion between ears. Traditional feature extraction methods struggle to specifically capture the directional features of wheat ears, such as growth angles and row arrangement directions, which makes the model prone to confusing targets with the background in complex scenarios, leading to false detections or missed detections of inclined wheat ears. The ordinary convolution operations in the YOLOv11 basic framework lack selective attention to direction-sensitive features, while the conventional ECA attention mechanism (Jiang et al., 2025; Qiong et al., 2025; Wang et al., 2020), although capable of learning channel dependencies through one-dimensional convolution, does not consider the spatial direction information of features, making it difficult to adapt to the morphological characteristics of wheat ears.

In view of this, this study proposes a direction-aware Oriented ECA attention mechanism. On the basis of retaining the efficiency of ECA, a direction feature decomposition and fusion strategy is introduced to enhance the model’s ability to capture the directional features of wheat ears. This mechanism first performs direction feature separation. Aiming at the horizontal and vertical distribution characteristics of wheat ears, two adaptive pooling operations are used to extract direction-sensitive features: horizontal direction pooling (avg_pool_h) compresses the width dimension of the feature map (AdaptiveAvgPool2d((None, 1))), retains the spatial distribution information in the height direction, and focuses on capturing the morphological features of wheat ears in the vertical dimension, such as ear length and inclination angle; vertical direction pooling (avg_pool_v) compresses the height dimension of the feature map (AdaptiveAvgPool2d((1, None))), retains the spatial distribution information in the width direction, and focuses on capturing the arrangement features of wheat ears in the horizontal dimension, such as row spacing and density.

Next, direction-dependent learning is carried out. The separated horizontal and vertical features are respectively subjected to one-dimensional convolution (conv_h and conv_v) to learn the feature dependencies within the directions: after dimension reorganization ([bh, 1, c]), the horizontal direction features capture the feature correlation at different height positions through one-dimensional convolution, enhancing the contour perception of inclined wheat ears; after dimension reorganization ([bw, 1, c]), the vertical direction features capture the feature correlation at different width positions through one-dimensional convolution, enhancing the ability to distinguish rows and columns of dense wheat ears.

Finally, direction attention fusion is performed. The attention weights of the horizontal and vertical directions (attn_h and attn_v) are element-wise added, and after Sigmoid activation, the final direction-channel joint attention map is generated. This attention map can not only adaptively adjust the channel weights, inheriting the efficiency of ECA, but also through the differential allocation of spatial direction weights, make the model prioritize attention to wheat ear regions with significant direction features, such as ears with matching inclination angles and continuous ear rows in dense arrangements.

Through the above design, the direction-aware Oriented ECA mechanism realizes the joint modeling of “channel importance” and “direction significance” of wheat ears. It not only avoids the neglect of spatial information by traditional channel attention, but also specifically optimizes the feature extraction ability for long strip and directional targets, providing more discriminative wheat ear feature representations for the subsequent detection head.

#### 2.3.3 Slim-neck

Traditional YOLOv11’s feature fusion layer fuses multi-scale features through simple concatenation or summation, failing to fully model cross-scale feature correlation. This approach is prone to causing key information loss, affecting target localization and classification capabilities. Meanwhile, its high computational cost and parameter count increase training time, storage burden, and may induce overfitting. To address these issues, this study employs the lightweight Slim-Neck module to reconstruct feature interaction paths, reducing model parameters while enhancing cross-scale aggregation of wheat ear features.

The core of the Slim-Neck (Li et al., 2022; Li et al., 2024; Huang et al., 2025) module lies in introducing an attention mechanism to adaptively weight feature maps of different scales, enabling the model to focus on important features and enhance feature representation. Attention sub-modules are established for each feature scale, which analyze channel and spatial dimensions to calculate feature weights and perform weighted fusion, effectively suppressing noise, highlighting key features, and improving wheat ear detection and recognition capabilities. Furthermore, this module adopts cross-scale feature interaction technology, designing special connections and convolution operations to establish multiple cross-scale connection paths between feature maps of different scales. This realizes bidirectional flow and complementarity between shallow and deep features, fully leveraging the advantages of features at different scales to improve the model’s detection performance for wheat ears of varying sizes and shapes.

**Figure 5:**
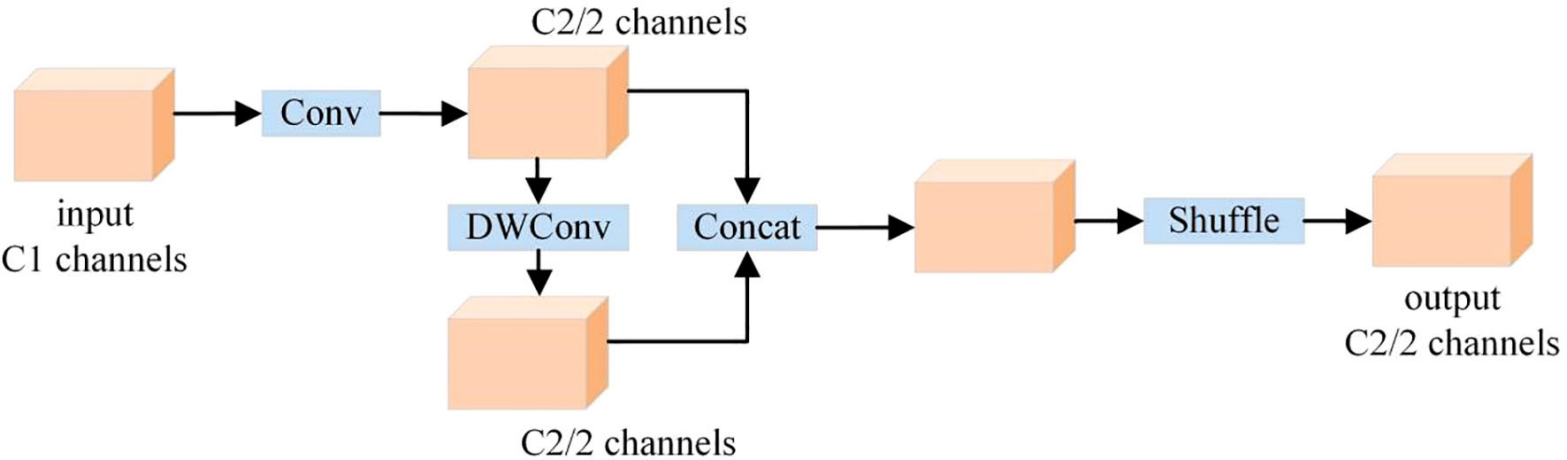
GSConv structure.

GSConv is a novel lightweight convolutional structure, whose structure is shown in **Figure 5**. It integrates standard convolution with depthwise convolution, a fusion approach that reduces redundant calculations in convolution operations and optimizes the data processing flow. This integration thereby significantly improves the convergence speed during the model training phase and the real-time response capability during the inference phase, enabling the model to complete object recognition tasks with higher efficiency while ensuring recognition accuracy.

### 2.4 Evaluation metrics

To test the improvement effects of this study, evaluation metrics such as Precision (P), Recall (R), and mean Average Precision (mAP) were used to assess the model performance. These metrics are defined in Equations 1-3 as follows:


(1) $$\text{P}=\frac{\text{TP}}{\text{TP}+\text{FP}}$$

(2) $$\text{R}=\frac{\text{TP}}{\text{TP}+\text{FN}}$$

(3) $$\text{mAP}=\frac{1}{\text{n}}\sum_{i=1}^{n}\int_{0}^{1}\text{P}(R)\text{d}(\text{R})$$

TP (True Positive) refers to the number of wheat ears correctly classified, i.e., the samples that are actually wheat ears and accurately detected by the model.

FN (False Negative) denotes the number of wheat ears misclassified, i.e., the samples that are actually wheat ears but missed (undetected) by the model.

FP (False Positive) represents the number of background regions incorrectly classified, i.e., the samples that are actually background but misidentified as wheat ears by the model.

AP (Average Precision) is the area under the precision-recall curve for the wheat ear category, quantifying the detection accuracy for single-category targets. Since wheat ears are the only detection target in this study, mAP (mean Average Precision) is equivalent to the AP value of this category. Specifically, mAP is typically the mean of AP values across all categories, but when only one category exists, its calculation simplifies to the AP of that single category.

In addition, when evaluating model performance, parameters (Params) and floating - point operations (FLOPs) are also important indicators. Parameters (Params) are static indicators representing the number of parameters contained in the model structure. They are determined before model training, usually measured in M (millions). The number of parameters reflects the size and complexity of the model. Floating - point operations (FLOPs), on the other hand, are dynamic indicators referring to the number of floating - point operations performed by the model during operation, mostly measured in G (gigabytes). Their values will vary with factors such as the size of the input data and the model’s inference process. Clearly distinguishing between these two indicators is of great significance for accurately evaluating the model’s computational resource requirements and performance.

## 3 Results and analysis

### 3.1 Experimental environment and parameter settings

The operational environment configuration for this study is as follows: the operating system is Windows 11, the PU is Intel(R) Core(TM) i7-14700KF, the GPU is NVIDIA GeForce RTX 4060 Ti with a video memory capacity of 8GB, and the RAM is 16GB. The deep learning framework used in the experiment is PyTorch (Version 2.5.0), CUDA (Version 12.4), and Python (Version 3. 10), with the programming environment being PyCharm. The basic parameters set for the experiments are as follows: the input image size is 640×640, the learning rate is 0.01, the batch size is 8, and the number of iterations (epochs) is set to 100.

### 3.2 Comparative experiments of different attention mechanisms

To improve key performance indicators such as model precision and recall, this study added four advanced attention mechanisms—CA (Ma et al., 2025), GAM (Li et al., 2023), CBAM (Woo et al., 2018), and OrientedECA—to the original YOLOv11 model for comparative detection performance experiments. The specific experimental results of different attention mechanisms are shown in **Table 2**. The results demonstrate that OrientedECA achieves the highest precision and recall without increasing model parameters or computational complexity, balancing model accuracy improvement and computational resource consumption.

**Table 2:** Performance comparison of different attention mechanisms.

Attention mechanism	P/%	R/%	mAP@0.5/%	mAP@0.5:0.95/%	Params/M	FLOPs/G
CA	91.6	90	95.2	53.6	2.6	6.3
GAM	90.5	90.4	95.7	52.7	4.2	7.6
CBAM	90.9	90.5	95.8	53. 1	2.6	6.4
OrientedECA	92. 2	91.6	95.9	53.6	2.6	6.3

### 3.3 Ablation experiments

To verify the improvement effects of various modifications in the YOLO v11-EDS network on model performance, multiple groups of ablation experiments were designed. The experimental results are detailed in **Table 3**.

**Table 3:** Ablation study results of different improvement methods.

OrientedECA	Dysample	SlimNeck	P/%	R/%	mAP@0.5/%	mAP@0.5:0.95/%	Params/M	FLOPs/G
×	×	×	91. 1	89.8	95.3	52.8	2.6	6.3
×	✓	✓	92.4	92. 1	96.8	54.4	2.5	5.8
✓	×	✓	91.5	92.8	96.7	54.2	2.5	5.8
✓	✓	×	92.7	92.6	96.7	54.4	2.6	6.3
✓	✓	✓	93.1	93.3	96.8	55.3	2.5	5.8

√indicates the use of this module; × indicates that the module is not used.

As shown in **Table 3**, the introduction of Dysample and SlimNeck modules improved precision by 1.3 percentage points, recall by 2.3 percentage points, mAP@0.5 by 1.5 percentage points, and mAP@0.5:0.95 by 1.6 percentage points. Meanwhile, model parameters were reduced by 0. 1M and floating-point operations (FLOPs) by 0.5G, indicating that these two modules enhance detection accuracy while achieving model lightweighting. When the OrientedECA and SlimNeck modules are introduced, the recall rate increases by 3.0 percentage points and mAP0.5:0.95 rises by 1.4 percentage points, which shows that OrientedECA enhances the adaptability to the morphology and growth angle of wheat ears by introducing the direction-aware mechanism, making feature extraction more in line with the target morphological characteristics, and especially improving the target recall effect in complex scenarios. When the OrientedECA and Dysample modules are added, the precision increases by 1.6 percentage points and mAP0.5 goes up by 1.4 percentage points, verifying that Dysample has the ability to retain detailed information during the feature map magnification process, and it forms a synergy with the direction-aware mechanism of OrientedECA, significantly improving the detection accuracy. When all three improved modules were used simultaneously, the model achieved optimal performance: precision increased by 2.0 percentage points, recall by 3.5 percentage points, mAP@0.5 by 1.5 percentage points, and mAP@0.5:0.95 by 2.5 percentage points. With parameters and FLOPs of 2.5M and 5.8G, respectively, the model reduced parameters by 0.1M and FLOPs by 0.5G compared to the baseline, achieving dual optimization of detection accuracy and inference efficiency. Experimental results show that all three proposed improvements effectively enhance model performance. The OrientedECA attention mechanism and Dysample upsampling operator significantly improve detection accuracy, while the SlimNeck module achieves model lightweighting while optimizing feature fusion and transmission. Their combination significantly improves the detection accuracy and inference efficiency of YOLOv11 for wheat ears in complex farmland environments, verifying the effectiveness and complementarity of each improved module.

### 3.4 Heatmap visualization

**Figure 6:**
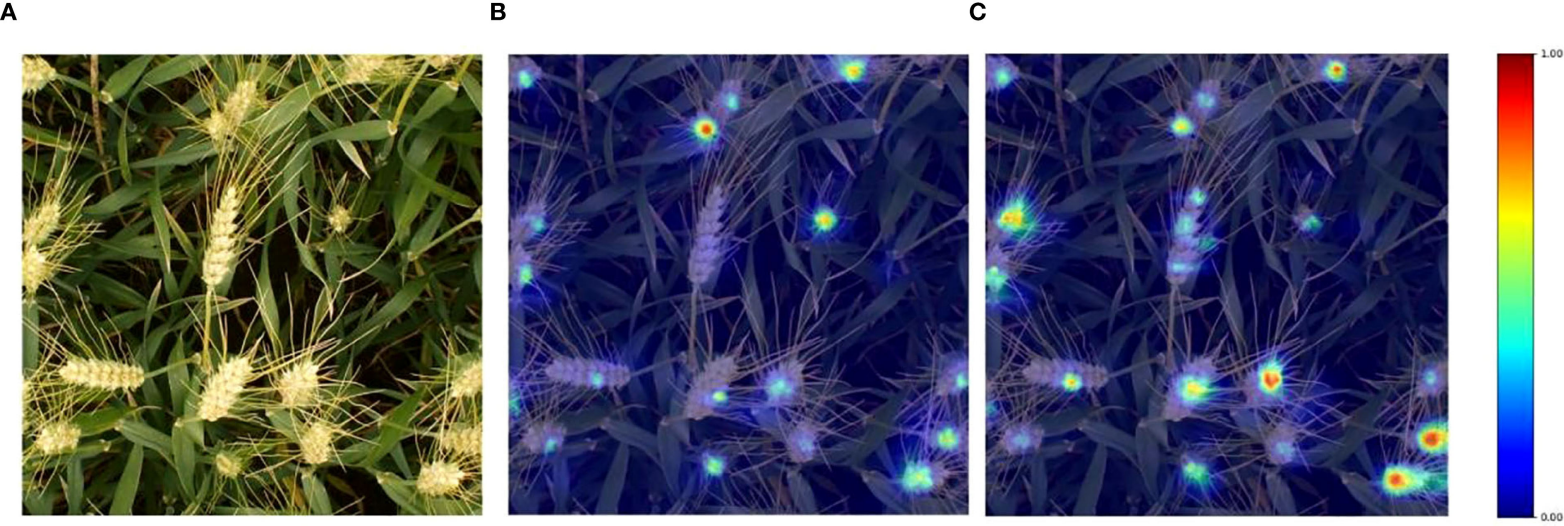
Visualization results of wheat ear image heatmap. **(A)** Original image; **(B)** YOLOv11; **(C)** YOLOv11-EDS.

Heatmap visualization serves as an important intuitive means to present the model’s learning process and analysis results. With the aid of HiResCAM heatmaps, it is possible to clearly gain insight into the model’s sensitive regions to input data and its internal operation mechanisms. As shown in **Figure 6**, taking an image from the wheat ear dataset as an example, the heatmap generated by the YOLOv11 baseline model shows that the heat values in some wheat ear regions are relatively low, reflecting that the model does not pay sufficient attention to these ears. In contrast, the heatmap of the improved YOLOv11-EDS significantly enhances the focusing ability on wheat ear targets, with heat distribution showing a higher degree of fit to the actual ear regions. This indicates that the improved model can extract more discriminative target features from input information, enabling more accurate detection of wheat ears under complex backgrounds. It effectively enhances the capability to capture ear targets, reduces potential missed detections, and fully demonstrates the advantages of the improved model in terms of robustness and generalization ability. The model can more efficiently focus on key target regions of wheat ears, thereby optimizing detection performance.

### 3.5 Comparative experiments of different algorithms

To compare the detection performance of the improved model with current mainstream object detection models for wheat ears, algorithms including Faster-RCNN (Sun et al., 2018), RT-DETR (Kong et al., 2024), RetinaNet (Wang et al., 2019), SSD (Liu et al., 2016), YOLOv5 (Tan et al., 2025), YOLOv8 (Li et al., 2024), and YOLOv11 (Khanam and Hussain, 2024) were used to train and test on the wheat ear dataset.

**Table 4** systematically compares the core performance indicators of different models in the wheat - ear detection task. The baseline model YOLOv11 demonstrates excellent comprehensive performance: with a precision of 91.1%, a recall of 89.8%, and an mAP@0.5 as high as 95.3%, it only requires 2.6M parameters and 6.3G floating - point operations. The performance of this model is significantly better than that of traditional detection models. Its mAP@0.5 is 16.8 percentage points higher than the 78.5% of Faster - RCNN, while the number of parameters is reduced by 34.1M and the computational load is reduced by 189.3G. Among the YOLO series, the detection accuracy of YOLOv11 is also leading. Its mAP@0.5 is approximately 1.9 percentage points higher than the 93.4% of YOLOv5 and approximately 1.0 percentage point higher than the 94.3% of YOLOv8.

**Table 4:** Experimental results of different models.

Model	P/%	R/%	mAP@0.5/%	Params/M	FLOPs/G
Faster-RCNN	76.3	81.2	78.5	36.7	195.6
RT-DETR	84.7	76.2	81.3	21.3	57.8
RetinaNet	85.3	81.5	84.2	35.3	241.4
SSD	80.4	79.6	80.1	26.5	92.3
YOLOv5	90.3	88.6	93.4	7.0	15.8
YOLOv8	91.2	89.5	94.3	3.0	8.1
YOLOv11	91.1	89.8	95.3	2.6	6.3
YOLOv11-EDS	93.1	93.3	96.8	2.5	5.8

The improved model YOLOv11 - EDS proposed in this paper achieves all - round performance breakthroughs: the precision is increased to 93.1%, the recall is increased to 93.3%, and the mAP@0.5 reaches 96.8%. The three key indicators are improved by 2.0, 3.5, and 1.5 percentage points respectively compared with the baseline model. In terms of model efficiency, the number of parameters is reduced to 2.5M, and the computational amount is compressed to 5.8G, achieving the simultaneous optimization of accuracy and efficiency. Horizontal comparison shows that the detection accuracy of this model significantly leads the mainstream detection frameworks: it is 12.6 percentage points higher than RetinaNet, 15.5 percentage points higher than RT - DETR, and also maintains a clear advantage in the YOLO series - 3.4 percentage points higher than YOLOv5 and 2.5 percentage points higher than YOLOv8.

The experimental verification shows that through the innovative feature - fusion architecture and lightweight design, YOLOv11 - EDS achieves higher wheat - ear detection accuracy in complex field scenarios while maintaining high - efficiency computing characteristics, providing an advanced solution with both high accuracy and low energy consumption for smart agriculture applications.

**Figure 7:**
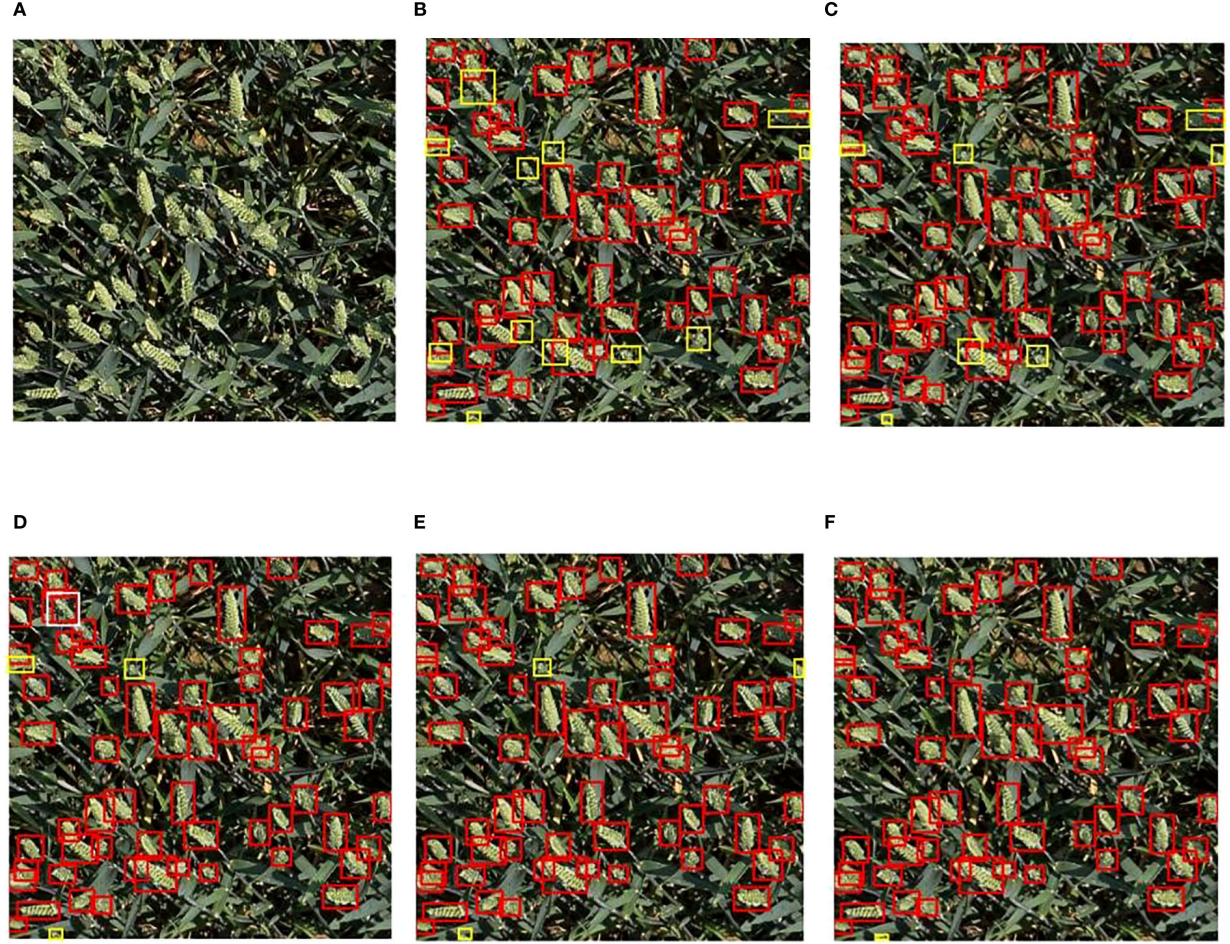
Comparison of detection performance of different models. **(A)** Original image; **(B)** Faster-RCNN; **(C)** YOLOv5; **(D)** YOLOv8; **(E)** YOLOv11; **(F)** YOLOv11-EDS. The red rectangular box represents the model prediction box, the yellow rectangular box represents wheat ears missed by the model, and the white rectangular boxes represent the redundant boxes generated by the model.

**Figure 7** presents a comparison of the performance of different models in detecting wheat ears in the field. Here, red rectangles represent the prediction boxes of the models, yellow rectangles mark the wheat ears missed by the models, and white rectangles indicate the wheat ears falsely detected by the models. It can be clearly seen from the figure that when wheat ears are highly similar to the complex background in terms of color and texture, YOLOv5 shows multiple yellow missed detection boxes in dense wheat ear areas. In particular, its ability to capture small-sized wheat ears is obviously insufficient, which fully reflects its weak ability to distinguish similar backgrounds. Although the detection performance of YOLOv8 and YOLOv11 has improved to some extent, there are still a small number of yellow missed detection boxes and white redundant boxes, which are mainly concentrated in areas where wheat ears overlap or are occluded. The YOLOv11-EDS proposed in this paper significantly reduces the number of missed detections. Especially in scenarios where wheat ears are densely arranged and the background is complex, it can still accurately distinguish the target from the background by virtue of efficient feature extraction capability, which strongly verifies the effectiveness of the improvement strategy.

### 3.6 Counting performance analysis

**Figure 8:**
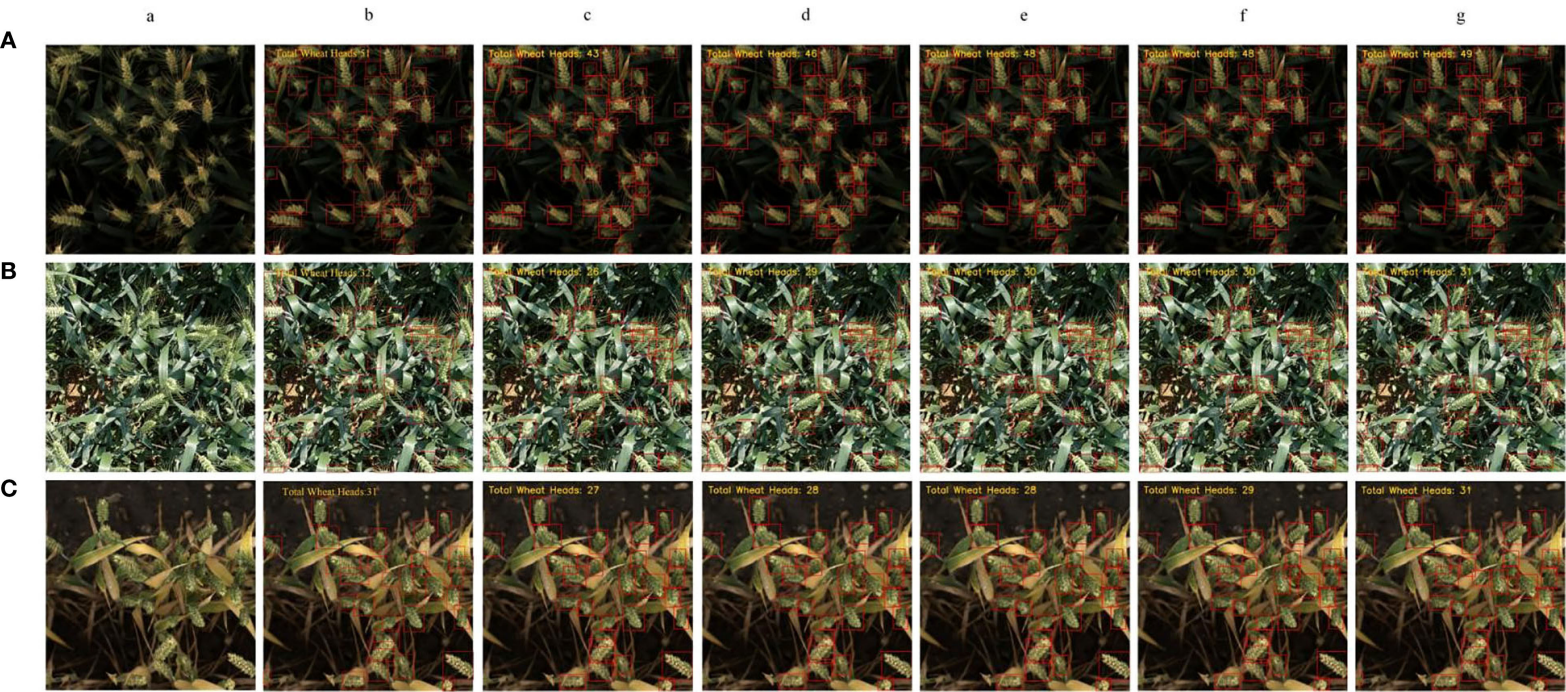
Comparison of counting performance of different models. **(A)** Low-light; **(B)** Strong-light; **(C)** Blurry; (a) Original images; (b) Ground truth; (c) Faster-RCNN; (d) YOLOv5; (e) YOLOv8; (f) YOLOv11; (g) YOLOv11-EDS.

**Figure 8** presents a comparative analysis of wheat ear counting performance by different models under three complex environments: strong light, low light, and blurry conditions. In the figure, bounding boxes annotate detected wheat ears, with manual counting results used as ground truth references.

Low-light environment: The ground truth count by manual counting was 51 wheat ears. Faster-RCNN, limited by low feature extraction efficiency, predicted 43 ears with 8 missed detections; YOLOv5 and YOLOv8 predicted 46 and 48 ears, with 5 and 3 missed detections, respectively; the baseline YOLOv11 predicted 48 ears with 3 misses; the improved YOLOv11-EDS, through enhanced feature extraction, predicted 49 ears with only 2 misses, showing the closest result to the ground truth.

Blurry environment: The manual counting ground truth was 31 ears. Faster-RCNN predicted 27 ears with 4 misses; both YOLOv5 and YOLOv8 predicted 28 ears with 3 misses each; YOLOv11 predicted 29 ears with 2 misses; YOLOv11-EDS accurately identified all targets, with the predicted count matching the ground truth—significantly outperforming other models.

Strong-light environment: The manual counting ground truth was 32 ears. Faster-RCNN, constrained by shallow feature extraction, predicted 26 ears with 6 misses; YOLOv5 predicted 29 ears with 3 misses; YOLOv8 and YOLOv11 predicted 30 ears with 2 misses each; YOLOv11-EDS predicted 31 ears with only 1 miss, exhibiting the smallest counting error.

Comprehensive experimental results show that YOLOv11-EDS, through multi-module collaborative optimization, effectively enhances the robustness of wheat ear detection in complex environments and demonstrates significant advantages in counting accuracy.

### 3.7 Model robustness validation on custom dataset

**Figure 9:**
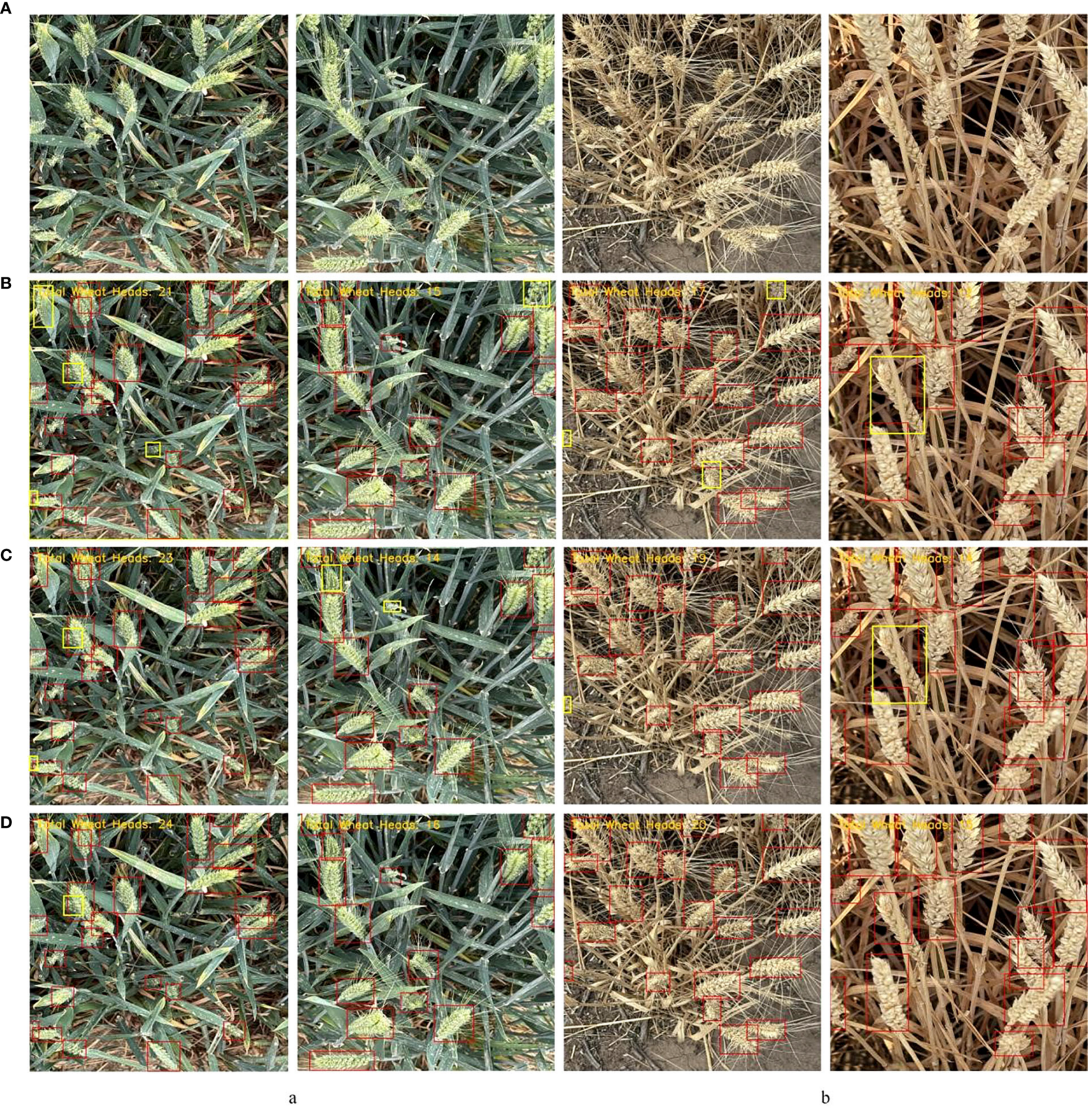
Detection visualization of self-built wheat ear dataset. **(A)**Original images; **(B)** RetinaNet; **(C)** YOLOv11; **(D)** YOLOv11-EDS; (a) Grouting period; (b) Maturity period. The red rectangular box represents the model prediction box, the yellow rectangular box represents wheat ears missed by the model.

To verify the effectiveness of the method proposed in this study, the YOLOv11-EDS model trained on the global wheat ear dataset was subjected to a robustness test using a self-built iPhone-perspective wheat ear dataset. **Figure 9** presents the visualization results of detection performance of different models on the self - built wheat ear dataset. Two typical images from the grouting stage and two from the maturity stage were selected to verify the robustness of the models. In terms of detection performance, RetinaNet showed a certain number of missed detections when dealing with wheat ears in complex scenarios, and its localization accuracy decreased especially in regions with densely arranged wheat ears or low color contrast. YOLOv11 exhibited a certain improvement compared with RetinaNet, being able to detect more wheat ear targets, but there is still room for improvement in the detection of small and occluded targets.

In contrast, relying on its unique architectural design and multi - scale feature fusion capability, YOLOv11 - EDS performed excellently in complex scenarios at different growth stages. Whether for plump wheat ears in the grouting stage or for the situation of reduced color contrast in the maturity stage, the model could locate and identify wheat ears relatively accurately, with the red prediction boxes highly fitting the main bodies of the wheat ears. Missed detections were mainly concentrated in extremely occluded regions, which indicates that YOLOv11 - EDS has good environmental adaptability and robustness in wheat ear detection tasks.

To further comprehensively evaluate the model performance, this study conducted a comparative analysis of three models, namely RetinaNet, YOLOv11, and YOLOv11-EDS, as shown in **Figure 9**. The evaluation adopted three indicators: mean absolute error (MAE), root mean squared error (RMSE), and coefficient of determination (R²). Among them, MAE reflects the average deviation degree between predicted values and true values, RMSE characterizes the dispersion of prediction results, and R² measures the goodness of fit of the trend line. All models were trained and tested on a self-built wheat ear dataset from an iPhone perspective, and the results are shown in **Table 5**.

**Table 5:** Comparison of counting performance metrics across models.

Model	MAE	RMSE	R²
RetinaNet	4.25	6.23	0.88
YOLOv11	3.68	4.96	0.92
YOLOv11-EDS	2.67	3.87	0.95

The analysis indicates that the YOLOv11-EDS model performed optimally in all indicators: it had the lowest MAE (2.67), the smallest RMSE (3.87), and the highest R² (0.95), which suggests that this model has the smallest prediction error, the most stable results, and the best goodness of fit. In contrast, all indicators of RetinaNet were relatively inferior, indicating that it has obvious limitations in processing wheat ear data from an iPhone perspective. Although YOLOv11 was superior to RetinaNet, there were still gaps with YOLOv11-EDS in terms of accuracy and goodness of fit. Overall, YOLOv11-EDS showed excellent performance stability in the wheat ear detection task from an iPhone perspective, providing a reliable technical basis for subsequent wheat ear phenotypic analysis and breeding evaluation.

**Figure 10:**
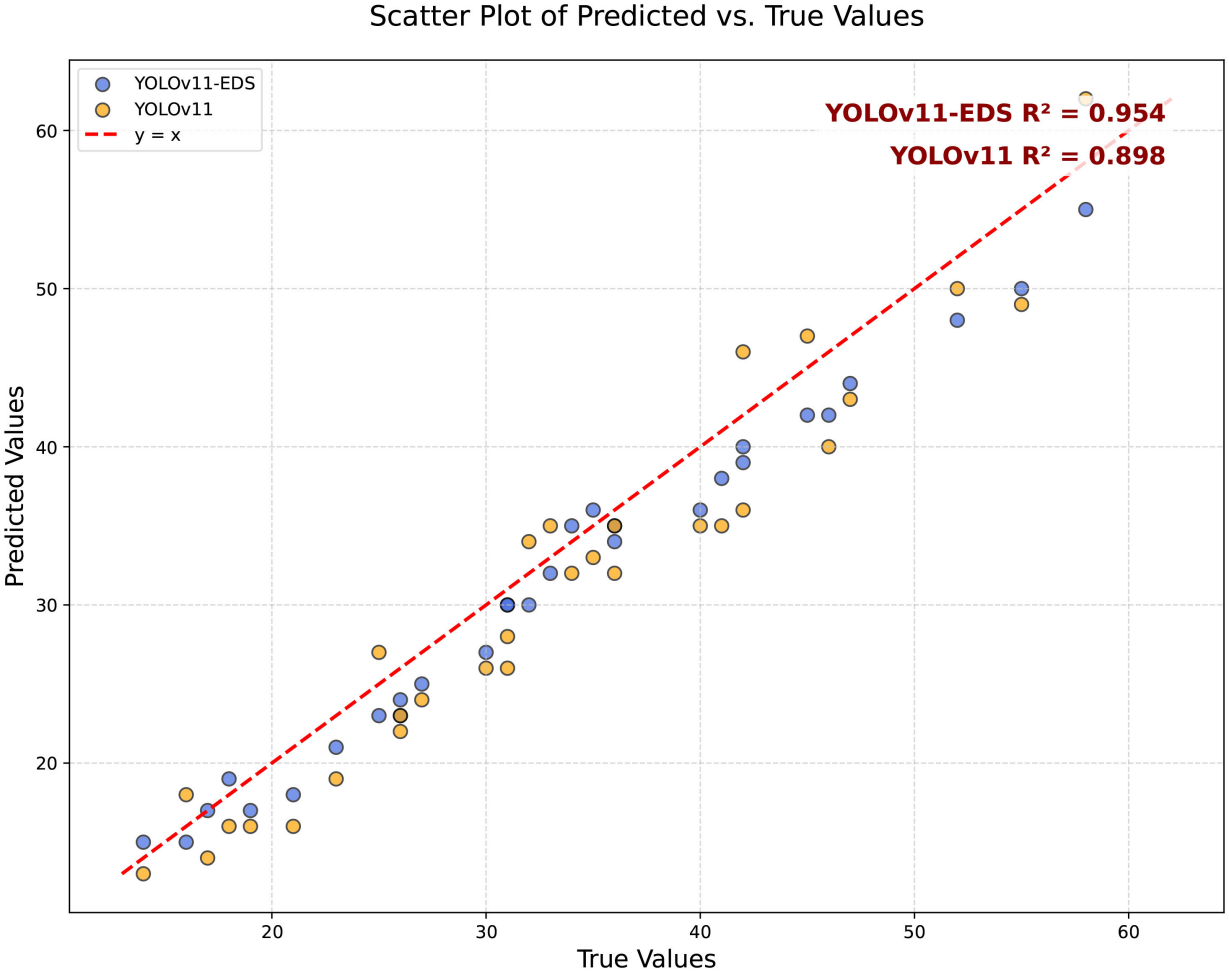
Scatter plot of predicted vs. true values of YOLOv11-EDS on the iPhone-view wheat ear dataset.

To further validate the counting accuracy of YOLOv11-EDS, this study randomly selected 30 representative samples from the dataset for in-depth analysis. To ensure the reliability and scientific rigor of the manual counting validation, a standardized verification process and evaluation criteria were established. The counting task was independently performed by three researchers with backgrounds in agronomy, all of whom received unified standardized training prior to the experiment. The training covered the identification of morphological characteristics of wheat ears at different growth stages and counting rules under complex conditions (such as occlusion and overlapping). The specific rules were as follows: (1) Occlusion handling rule: Only targets with a visible proportion exceeding 40% that could be unambiguously identified as independent ears were counted; targets that could not be reliably identified were excluded. (2) Overlapping and clustering rule: When multiple wheat ears overlapped but were distinguishable by contour, they were counted separately; if the ears were tightly clustered and could not be reliably separated, the entire cluster was counted as one unit. All counters independently completed the counting of all 30 images without knowledge of each other’s results, and the final manual counting ground truth was obtained by averaging the results from the three counters. The consistency of counting was quantitatively evaluated using the intraclass correlation coefficient (ICC = 0.962), which confirmed the high reliability of the manual ground truth. Based on the above standardized manual annotation results, the detection results of the YOLOv11-EDS and YOLOv11 models were quantitatively compared against the manual ground truth, as shown in **Figure 10**.

**Figure 11:**
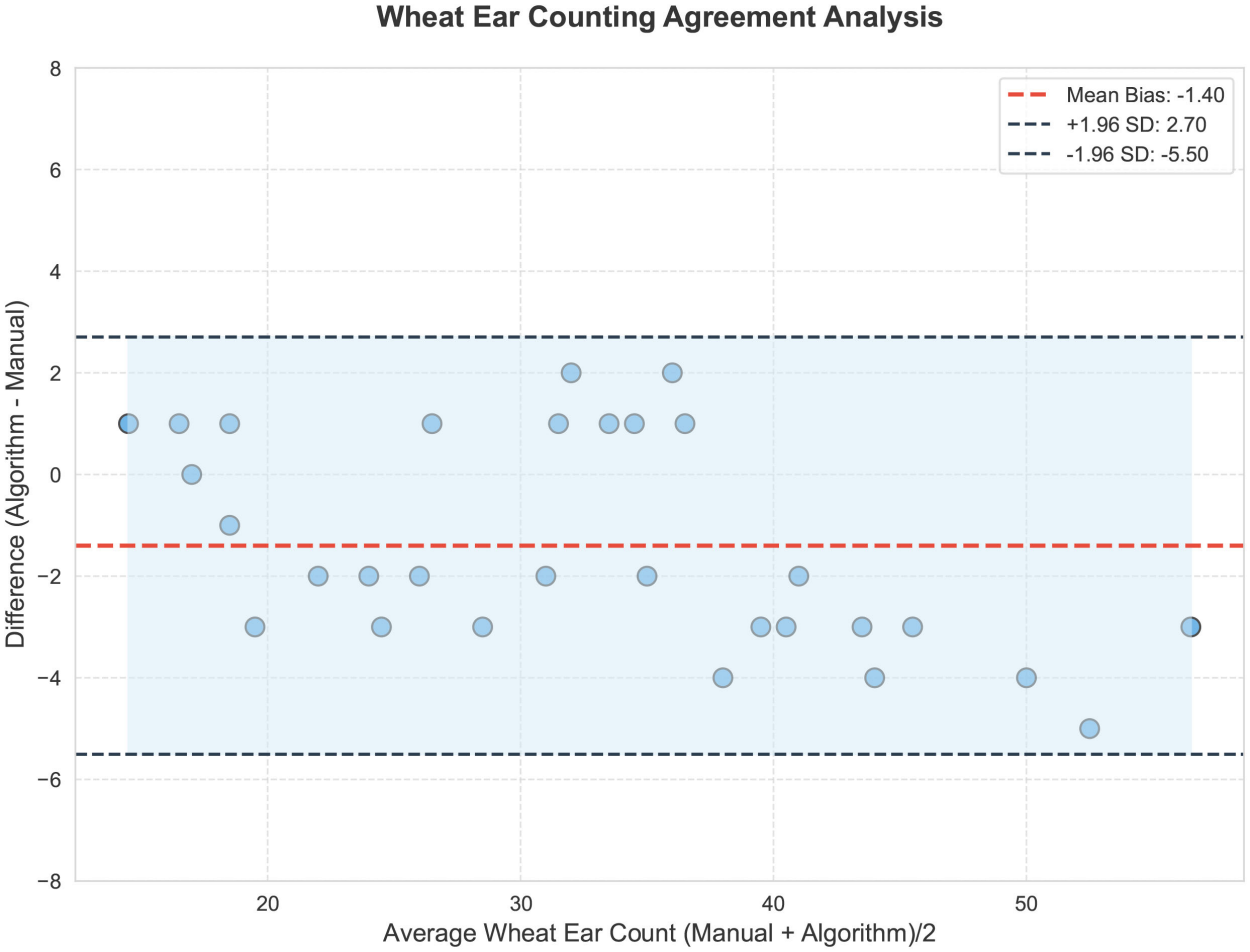
Bland - Altman analysis plot.

The YOLOv11-EDS model achieved a coefficient of determination (R²) of 0.954 between predicted and true values, with all data points closely distributed along the y=x reference line, visually confirming a strong agreement. In contrast, the original YOLOv11 model yielded an R² value of 0.898, with data points exhibiting a more scattered distribution. The experimental results demonstrate that the counting error of YOLOv11-EDS remains within an acceptable range even in complex field scenarios. Its robustness supports the requirement for automated wheat ear detection in the field, thereby providing a reliable technical foundation for yield estimation in precision agriculture **Figure 11** is a Bland - Altman analysis plot of the consistency between the model and manual annotations in wheat ear counting. The horizontal axis is “Average Wheat Ear Count (Manual + Algorithm)/2”, which is the average value of manual counts and model counts; the vertical axis is “Difference (Algorithm - Manual)”, that is, the difference between the model count and the manual count. The black dashed lines represent the limits of agreement (95% LoA), with an upper limit of 2.70 and a lower limit of -5.50, meaning that 95% of the data points should fall between these two lines. The light - blue translucent area corresponds to the 95% limits of agreement range, in which the scatter points are distributed. As can be seen from the figure, most of the scatter points are within the limits of agreement, and there is no obvious distribution trend, indicating that the counting results of the improved model YOLOv11 - EDS and manual annotations have good consistency in the wheat ear counting task. The model performance is relatively reliable, and it can count the number of wheat ears relatively accurately.

### 3.8 Occlusion robustness test

**Figure 12:**
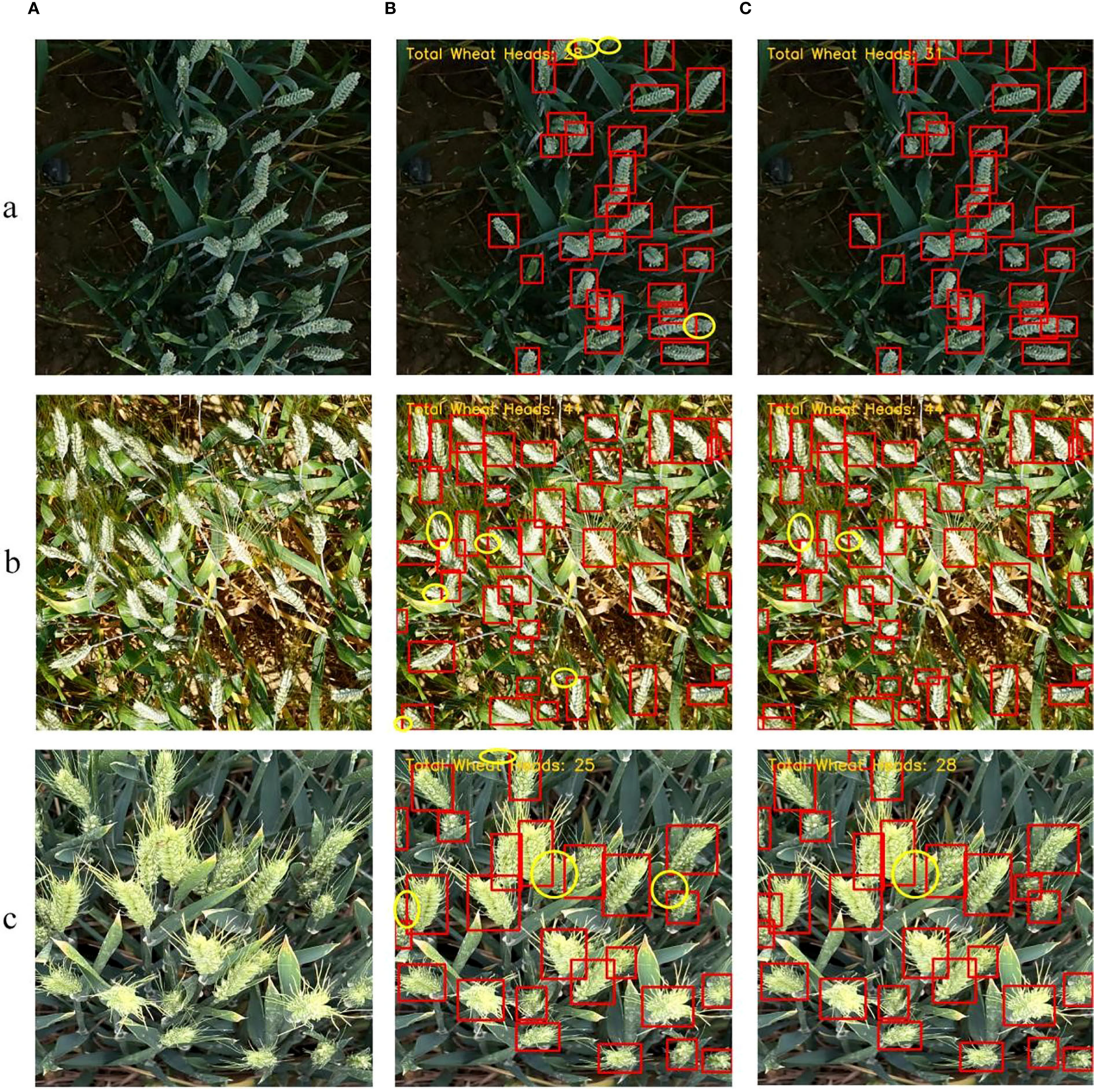
Detection results comparison between YOLOv11 and YOLOv11-EDS under occlusion conditions. **(A)** Original images; **(B)** YOLOv11; **(C)** YOLOv11-EDS; (a) Low light; (b) Strong light; (c) Normal light. Note: Red rectangles represent model prediction boxes, and yellow ellipses represent wheat ears missed by the model.

To validate the model’s performance under complex environments with varying lighting conditions and occlusion scenarios, we selected multiple sets of representative images from both public and self-built datasets. These images cover different lighting conditions such as low light, strong light, and normal light, and include moderate to severe occlusion phenomena. **Figure 12A** shows the original images, while **Figures 12B, C** present the detection results of YOLOv11 and YOLOv11-EDS, respectively, where (a) represents low light, (b) strong light, and (c) normal light. Red bounding boxes indicate the targets predicted by the models, and yellow elliptical boxes mark the missed wheat ears.

The experimental results demonstrate that under different lighting conditions and significant occlusion, YOLOv11 exhibited a considerable number of missed detections, as evidenced by the notable quantity of yellow elliptical boxes. In contrast, YOLOv11-EDS significantly reduced the number of missed detections across all lighting conditions, demonstrating superior adaptability to both lighting variations and occlusion. Furthermore, the improved model maintained strong detection performance for overlapping wheat ears, with only occasional missed detections in extreme occlusion cases (such as wheat ears obscured by leaves by more than 60%). These results verify that YOLOv11-ETS retains strong detection reliability even in complex occlusion scenarios, indicating that the proposed improvement strategies in this study effectively enhance the model’s occlusion robustness.

## 4 Discussion and conclusions

Accurate detection and counting of wheat ears are pivotal for wheat yield estimation and agricultural production management. To address challenges in complex field environments, this study proposes the YOLOv11-EDS model by refining YOLOv11, tailored for efficient wheat ear detection and counting.

1. Specifically, three key improvements were implemented: the Dysample dynamic upsampling operator was introduced to adaptively adjust the upsampling process based on diverse image features, enhancing feature map information transmission and strengthening the model’s capability to capture multi-scale wheat ear features. Additionally, Incorporated is the specially designed the Direction-aware Oriented Efficient Channel Attention (OrientedECA) module, which enhances adaptability to the morphology and growth angle of wheat ears by introducing a direction-aware mechanism, making feature extraction more in line with the target morphological characteristics. While guiding the model to focus on representative feature channels and adaptively reweighting channel-wise features, it strengthens the learning of key directional features of wheat ears. Finally, the Slim-Neck module was integrated to optimize the feature fusion structure, enabling more efficient integration of cross-layer feature information and boosting detection performance in complex scenarios.
2. Experimental results demonstrated significant performance advancements of YOLOv11-EDS: in terms of detection accuracy, P increased by 2.0 percentage points, R improved by 3.5 percentage points, mAP@0.5 rose by 1.5 percentage points, and mAP@0.5:0.95 climbed by 2.5 percentage points compared to the baseline YOLOv11, showcasing superior target recognition and recall capabilities. Concurrently, the model achieved effective lightweight optimization: parameter count was reduced to 2.5 M, and computational complexity (FLOPs) dropped to 5.8 G, ensuring robust detection performance while enabling deployment on resource-constrained devices.
3. Compared with other mainstream detection networks, YOLOv11-EDS exhibited superior comprehensive performance in detection accuracy, miss detection rate, and computational efficiency. Robustness testing on a self-built wheat ear dataset (captured from an iPhone perspective) confirmed stable detection performance under complex field conditions, providing reliable technical support for subsequent wheat yield estimation and promoting the intelligent transformation of agricultural production management. Future work will focus on optimizing the model’s adaptability to complex scenarios such as extreme occlusion, with particular emphasis on exploring the incorporation of explicit occlusion-handling mechanisms—such as partial convolution or attention modules—to further enhance its perception and recognition performance under occluded conditions. Meanwhile, we will investigate multimodal data fusion strategies to improve detection robustness and facilitate its wider application in intelligent wheat production systems.

## Data Availability

The raw data supporting the conclusions of this article will be made available by the authors, without undue reservation.
